# Digital Image Tamper Detection Technique Based on Spectrum Analysis of CFA Artifacts

**DOI:** 10.3390/s18092804

**Published:** 2018-08-25

**Authors:** Edgar González Fernández, Ana Lucila Sandoval Orozco, Luis Javier García Villalba, Julio Hernandez-Castro

**Affiliations:** 1 Group of Analysis, Security and Systems (GASS), Department of Software Engineering and Artificial Intelligence (DISIA), Faculty of Computer Science and Engineering, Office 431, Universidad Complutense de Madrid (UCM), Calle Profesor José García Santesmases 9, Ciudad Universitaria, 28040 Madrid, Spain; edggonza@ucm.es (E.G.F.); asandoval@fdi.ucm.es (A.L.S.O.); 2 School of Computing, Office S129A, University of Kent, Cornwallis South Building, Canterbury CT2 7NF, UK; J.C.Hernandez-Castro@kent.ac.uk

**Keywords:** Bayer Filter, CFA artifacts, Color Filter Array, Discrete Cosine Transform, Image Forensics, image tamper detection

## Abstract

Existence of mobile devices with high performance cameras and powerful image processing applications eases the alteration of digital images for malicious purposes. This work presents a new approach to detect digital image tamper detection technique based on CFA artifacts arising from the differences in the distribution of acquired and interpolated pixels. The experimental evidence supports the capabilities of the proposed method for detecting a broad range of manipulations, e.g., copy-move, resizing, rotation, filtering and colorization. This technique exhibits tampered areas by computing the probability of each pixel of being interpolated and then applying the DCT on small blocks of the probability map. The value of the coefficient for the highest frequency on each block is used to decide whether the analyzed region has been tampered or not. The results shown here were obtained from tests made on a publicly available dataset of tampered images for forensic analysis. Affected zones are clearly highlighted if the method detects CFA inconsistencies. The analysis can be considered successful if the modified zone, or an important part of it, is accurately detected. By analizing a publicly available dataset with images modified with different methods we reach an 86% of accuracy, which provides a good result for a method that does not require previous training.

## 1. Introduction

Nowadays technology allows inexperienced users to create and modify digital media with little effort. This is helpful in many areas that require sophisticated techniques to improve digital content. However, malicious entities can take advantage of this tools to create false information. It is difficult for audience with no skills in the area to conclude if the observed media is authentic or not. This can influence decisions that might affect several entities, say individuals, corporations or government institutions. To provide certainty that the examined information is true, several digital forensic techniques have been developed in recent years [1,2].

In this work, a new method for image tampering detection on images that exhibit Color Filter Array (CFA) artifacts is provided in detail. This method does not require previous training or knowledge of the source, offering a good option for quick forensic analysis. Since analysis can be done even on small blocks of size 2 × 2, fine grained analysis is supported, and even small modifications can be detected.

### Digital Image Processing

We briefly mention the process of creation of digital images in cameras having CFA sensors, which are very common in the digital camera industry.

Sensors using CFA technology are widely used in the photographic industry. Most commercial products in the market use either charge-coupled device (CCD) or complementary metal-oxide-semiconductors (CMOS). They work in a similar way, although the key difference is in the way in which pixels are scanned and the way in which the reading of the charges is carried out. In both cases, the image sensor is an array of light sensitive elements called pixels, which are made of silicon. In CCD sensors, information measured by each cell is transformed into voltage, resulting in an analog signal that will be digitized later by the camera. Pixels in a CCD array capture light simultaneously, providing a more uniform output, but a major drawback is the need of an additional device to process the sensor’s output information, which results in more costly and bigger digital cameras. On the other hand, CMOS sensors have an independent and active pixels design. Pixels are provided with capacitors and amplifiers, thus digitization can be done internally, offering better processing speed and eliminating the need for an external chip as in the case of CCD, reducing cost and size of equipment. Pixels transform incoming light converting photons into charge carriers by means of the photoelectric effect. Each pixel accumulates the charge induced by the light during the exposure time until it is read and processed. The output signal is proportional to the accumulated charge, depending on the amount of light captured by the pixel and the exposure.

**Figure 1:**
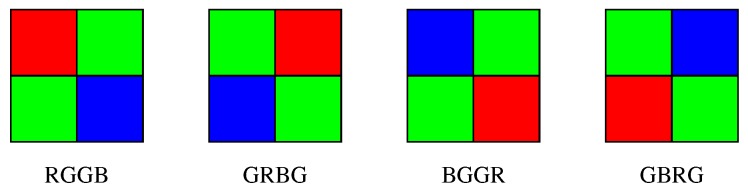
Different configurations of Bayer Filters.

The sensor splits the incoming light in different wavelengths or colors , which are joined together in the post-processing phase to create the color digital image. Ideally, a sensor should be used for each wavelength, but the cost of these electronic devices results in more expensive photographic devices. Therefore, it is common to use filters that capture different color ranges. So far, the proposed technique has performed pretty well on one the most common color filters, the Bayer Filter, which filters light in three different colors: blue, green and red. Four different configurations of this filter are depicted in Figure 1, all of them consisting of a matrix array composed of 2 green, 1 blue and 1 red pixels. However, many other configurations using different color positions or different wavelengths, as those found in [3,4], can be considered.

The use of this type of color filters in photographic cameras results in inconsistencies in the estimated pixels due to the application of different methods. In the case of images created from CFA devices, correlations created from demosaic algorithms [5,6,7,8] can be detected and used to exhibit tampering. Pixels captured directly by the sensor have a higher variance, unlike values estimated through interpolation techniques.

In addition to imperfections caused by interpolation algorithms, noise can be introduced at different phases of the processing pipeline of the image. Photo Response Non-Uniformity (PRNU) is the most predominant part of the noise, and it it is mainly caused by hardware defects. PRNU noise is composed by the Pixel Non-Uniformity (PNU) and the low-frequency defects. The former consists in the difference in sensitivity of pixels to light due to manufacturing imperfections. The later is caused by zoom settings, light refraction, dust particles, and lenses. This anomalies in the process of image creation are also exploited to provide tamper detection techniques or source identification [9,10,11]. Part of the PRNU noise is consistent among captures made with the same device, and traces of the pattern left by the fixed noise can be used as a fingerprint for the source device if it can be measured accurately, but it requires of a large set of samples from the device and previous training to get the exact pattern.

**Figure 2:**
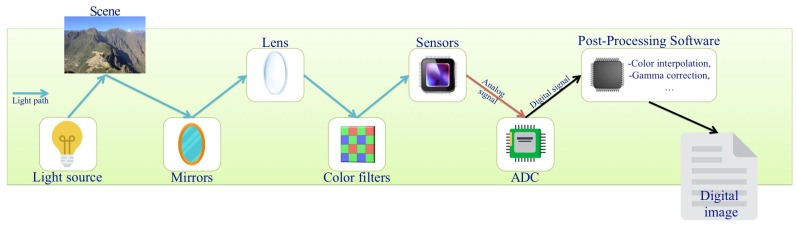
Image formation process. CFA interpolation artifacts are introduced by color filters.

Detailed information of photo processing methods are in general not easily available, and so it is not possible to analyze images accurately even with knowledge of the camera brand and model. Furthermore, some of these processes can destroy the CFA artifacts of interest in this study. For instance, smoothing filters, which are used to reduce the impact of noise on images, and this can well affect the behaviour of artifacts. Also compression can destroy some evidence of how the image has been initially constructed. A detailed model of the sensor output is provided in [12]. A picture of a simplified process of image formation can be seen in Figure 2.

The remaining sections of the present work are divided as follows: a brief summary of related techniques is presented in Section 2. Later, in Section 3, Ordinary Least Squares (OLS) process for kernel estimation is briefly addressed to obtain the residual matrix. Section 4 explains the process of computing the probability map from the residuals, and how this map is transformed using the Discrete Cosine Transform (DCT) to exhibit tampering. Experimental results obtained in images from the dataset provided in [13] are presented in Section 5. Finally, Section 6 shows the main conclusions and future work.

## 2. Previous Work

Let us denote the set of acquired and interpolated pixels of a Bayer Filter with A and I respectively. The presence of two different distributions in images obtained from a Bayer Filter is studied in [14]. From an estimation of the elements in I, the local variance of residuals is calculated taking into account the errors of the closest *N*-neighbors. Subsequently, a characteristic value is extracted in blocks of size *B* considering the ratio of the residuals variance for I and A. According to [15], the variance of the elements in I is smaller than variance of those in A. It is assumed that a modification has been done if this does not happen.

In [16], the evidence of image tampering is suggested by quantifying correlations introduced by the CFA interpolation. First an estimation of the interpolation kernel is carried out by using the Expectation-Maximization (EM) algorithm. While performing this estimation, a probability map is computed and used to identify interpolated from acquired pixels by assigning to the pixel in the position (x,y) the value determined from a mixture of Gaussian and Uniform distributions as seen in (1), where $\phi_{\mu ,\sigma }$ corresponds to the normal density with mean μ and standard deviation σ.

(1) $$P\left(x,y\right)=\frac{\phi_{\mu ,\sigma }\left(x,y\right)}{\phi_{\mu ,\sigma }\left(x,y\right)+\frac{1}{256}}$$

Subsequently, a measure of similarity between the obtained probability map and a synthetic map representing the positions of interpolated and acquired pixels is obtained from the magnitude of the Discrete Fourier Transform (DFT) of the probability map. Though effective to detect localized tampering, the EM step is computationally expensive and does not take in account the assumption that the image comes from a device with a CFA sensor. However, as seen in more detail in [17], this technique can be useful to detect modifications and provide an estimate for the transformation applied on the whole image, like resizing and rotation.

The contribution introduced in [18] consists of two steps. First a noise extraction is performed by means of the Discrete Wavelet Transform DWT. It is expected that CFA artifacts coming from distributions errors are exposed in this step. A feature is proposed by defining a feature on small blocks of the image noise, which depends on the ratio between interpolated and acquired pixels. The definition of the recommended feature is as follows

(2) $$F_{2}=\max\left(\frac{var(A_{1})}{var(A_{2})},\frac{var(A_{2})}{var(A_{1})}\right),$$

where $A_{1}$ y $A_{2}$ are the values of the noise in the interpolated and non-interpolated pixels respectively. Values close to 1 show possible manipulations in the inspected block.

Copy-move forgeries consist of copying small parts of an image that will be pasted within the same image. The copied areas can be modified by using filters, geometrical transformations (resize, rotate) or color changed. The most simple case, consisting of only translating the copied area, has been effectively detected in [19,20]. The detection technique consists in computing the DCT coefficients on overlapping blocks. Then the DCT coefficients are considered as 1-D vectors and ordered using lexicographic ordering. For each vector, a set of its *n* closest neighbors are compared and similar blocks will be linked by a transfer vector, which is the difference the position of the upper-left pixel of the related blocks. If enough blocks are found with the same transfer vector, they are considered as part of the manipulated zone. More complex modifications involving resize and rotate transformations are approached in [21,22,23], where the Scale-Invariant Feature Transform (SIFT) is used. This technique consists in the detection of points of interests, like corners, edges or texture. Additional techniques developed in [24] can be used to detect this manipulations.

Color change manipulations can be achieved by modifying the color in a section of the image. Detection techniques involving CFA artifacts are provided in [25,26]. The modification is exposed by analyzing statistical inconsistencies for the hue and saturation channels after colorization.

Additional to CFA artifacts, the PRNU noise has been utilized as a source of information to detect forgeries. Noise produced by manufacturing defects or hardware damage is consistent among images coming from the same device, thus any picture showing an uncorrelated PRNU pattern can be classified as tampered. A common measure for the pattern matching is based on correlation between noise samples. In [13,27] this approach is studied, and a null hypothesis is performed to decide whether an image has been tampered or not.

Though similar to what is done in [16], the probability map introduced in the present work and the inspection of the DCT coefficients provide better results for small blocks in the tests accomplished so far, getting good results even for blocks of size 2 × 2, suitable for detection of small alterations as will be discussed in more detail in Section 4 and Section 5. Furthermore, the approach of using the DFT magnitude to measure the similarity has a major flaw. If the CFA pattern of a section in the image is reversed as a result of a modification (e.g., reflection, rotation or copy-move), the DCT coefficient here considered is negated, in contrast to the magnitude of the DFT coefficient, which does not change in this case. The same issue can be found in [18], where the feature $F_{2}$ only detects an illegal modification when the difference of the variance between errors from acquired and interpolated pixels diminishes.

Small modifications are also detected in [13,27], but previous training is needed to get an estimation of the PRNU noise of a camera, which makes our method suitable if the device is not available to perform an accurate noise extraction. As it will be mention in Section 5, some instances of the copy-move forgery can be detected if the original CFA pattern is broken. Thus the application of resizing, rotation or reflection on the copied area makes the detection easier, which is not the case when only a translation is carried out. This case can be easily solved by using the technique proposed in [19,20], which does not need the hypothesis here required concerning the image formation from a CFA filter. Finally, colorization alterations found in the studied dataset have been detected, but this has not been deeply studied with the method proposed here. Further methods for colorization detection aforementioned should be used together with the process to be described for better results.

## 3. Interpolation Kernel Estimation

The method used for the interpolation kernel estimation is a simply OLS regression applied on the set of *N*-neighbors of each pixel given that this approach provides good results. The details of this technique applied to the green channel of an image is detailed next.

**Figure 3:**
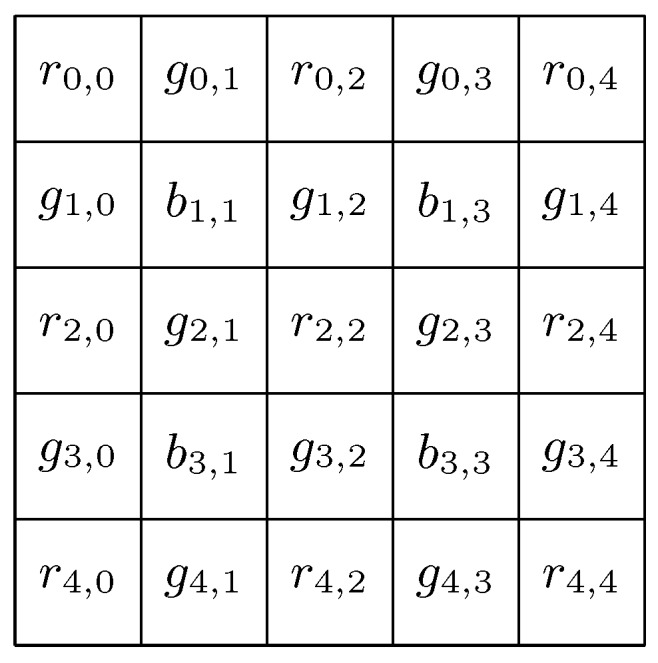
Bayer Filter values.

Consider a Bayer Filter *C* as shown in Figure 3 and let *G* be the green channel matrix built from *C* as shown in (3).

(3) $$G\left(i,j\right)=\left\{\begin{array}{cc} C(i,j) & \mathrm{if}\;i+j\;\mathrm{is}\;\mathrm{odd}\\ 0 & \mathrm{otherwise} \end{array}\right.$$

It is assumed that the interpolation method is given by

(4) $${\hat{g}}_{i,j}=\sum_{m=-N}^{N}\sum_{n=-N}^{N}h_{m,n}g_{i+m,j+n}.$$

with $h_{m,n}\in R$ for m,n=−N,…,N. By assuming that only values from *G* at positions (i,j) with i+j odd are used to resample the green channel matrix, we can get rid of half of the independent variables to perform OLS. Then, from the ${\left(2*N+1\right)}^{2}$ values $h_{m,n}$, those having indices (m,n) with m+n odd do not modify the final result since G(i,j) is 0 at those positions, so an arbitrary value can be assigned. Usually, demosaic algorithms leave values of acquired pixel unchanged, so these are set to zero. For the analysis to be performed, both, acquired and interpolated pixels will be reestimated with the values of the green channel, then $h_{0,0}$ is also set to zero.

Although more sophisticated interpolation methods that consider the other bands, such as those proposed in [6], for simplicity in the proposed method and due to the good results obtained in the experiments, only green channel values are considered in the analysis. To better understand the estimation process, the OLS method for N=1 is detailed next. Considering the Bayer Filter with elements as shown in Figure 3, estimation of the value $g_{2,2}$ is shown in (5).

(5) $${\hat{g}}_{2,2}=h_{1,2}g_{1,2}+h_{2,1}g_{2,1}+h_{2,3}g_{2,3}+h_{3,2}g_{3,2}$$

An approximation $h_{i,j}^{\prime }$ has to be done in such a way that the residuals of the values interpolated with (4) are as small as possible. This is, we need to reduce the value of (6) as possible.

(6) $$\epsilon_{i,j}={\hat{g}}_{i,j}-g_{i,j}^{\prime }$$

For this, these coefficients are approximated by applying OLS where the dependent variable *Y* is the set of interpolated values and the independent variables $X_{i}$ are the corresponding *N*-neighbors. Thus, the matrices used in the estimation from the matrix of interpolated values $\hat{G}$ will have the form presented in (7).

(7) $$Y=\left(\begin{array}{c} {\hat{g}}_{1,1}\\ {\hat{g}}_{1,2}\\ {\hat{g}}_{2,2}\\ {\hat{g}}_{3,1}\\ {\hat{g}}_{3,3} \end{array}\right),X=\left(\begin{array}{c} g_{0,1},g_{1,0},g_{1,2},g_{2,1}\\ g_{0,3},g_{1,2},g_{1,4},g_{2,3}\\ g_{1,2},g_{2,1},g_{2,3},g_{3,2}\\ g_{2,3},g_{3,2},g_{3,4},g_{4,3}\\ g_{2,3},g_{3,2},g_{3,4},g_{4,3} \end{array}\right).$$

The value of the coefficient vector $h^{\prime }$ that minimizes the sum of squares of the residuals $\sum_{i,j}\epsilon_{i,j}^{2}$ is given by (8).

(8) $$h^{\prime }={\left(X^{T}X\right)}^{-1}X^{T}Y.$$

Employing matrix notation, the set of coefficients is organized and written as shown in (9).

(9) $$H^{\prime }=\left(\begin{array}{ccc} 0 & h_{1,2}^{\prime } & 0\\ h_{1,4}^{\prime } & 0 & h_{2,1}^{\prime }\\ 0 & h_{2,3}^{\prime } & 0 \end{array}\right).$$

The approximation $G^{\prime }$ obtained by applying the interpolation can be expressed as the convolution $G^{\prime }=H^{\prime }⋆\hat{G}$. It should be noted that with this method the sum of the quadratic residuals of the interpolated values is reduced to a minimum, since the acquired values are not taken into account.

This method can be used for any value of *N* by choosing blocks of size ${\left(2N+1\right)}^{2}$ and extracting the acquired values to make the rows of the matrix *X*. The number of columns is determined by $\frac{{\left(2N+1\right)}^{2}-1}{2}$, since only half of the values of neighbors in this block belong to the set of acquired pixels. The number of rows will depend on the size of the image to be analyzed.

It is been observed that in many cases this first step can be avoided since direct use of simple interpolation methods, e.g., bilinear or bicubic, does not change the accuracy of the test.

## 4. CFA Tamper Detection with DCT

In order to perform a statistically significant analysis, it is important to make a good estimation of the interpolation coefficients. The distribution of the errors gives a good indicator of the goodness of fit of the kernel estimation. However, as indicated in Section 3, kernel estimation step can be ignored if simpler methods behave well. In order for this test to be successful, an initial hypothesis, regarding the distribution of the residuals of estimation for the sets I and A must be fulfilled, otherwise our method has poor chances of succeeding.

The presented contribution consists mainly of 4 steps:

1. Assuming that the configuration of the CFA pattern is known, a simple estimation of the interpolation kernel for the green channel is generated based on OLS. Only acquired pixels will be considered to get a better estimation.
2. An estimation of the image is obtained by using the interpolation kernel computed in the previous step on every pixel. Then, from the residuals between the estimation and the original image the standard deviation for interpolated and acquired pixels is computed. According to [15], and denoting by $\sigma_{I},\sigma_{A}$ to the standard deviation for interpolated and acquired residuals respectively, it should be observed that $\sigma_{I}\approx \frac{\sigma_{A}}{2}$.
3. Similar to what is done in [16], a probability map is generated to decide if a pixel belongs to the set of resampled data. However, the complementary error function is used, which defines the probability of a pixel of lying in the set I.
4. Finally, the DCT is applied on blocks of size B×B to verify the presence of the CFA artifacts within the block. The DCT coefficient for the highest frequency is considered as an indicator to detect of tampering. Unusual values (lower or higher than expected) in the coefficient for the highest frequency after applying DCT is used as evidence of modification of the image.

The details of the computation for the probability map and the DCT coefficient extraction is detailed next.

### 4.1. Probability Map

As previously mentioned, in an image created from a Bayer Filter, standard deviation of acquired pixels will be higher than deviation from interpolated ones. This information will be useful in the detection of inconsistencies by creating a probability function to decide whether a pixel belongs to the set I or a sample with a different distribution. In general green channel analysis is sufficient to detect tampering, so a detailed analysis of the process on this channel is given next.

**Figure 4:**
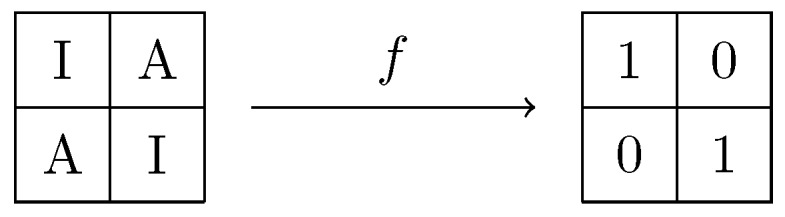
Classification of pixels using a probability function.

First, previous knowledge of the Bayer Filter configuration is assumed to extract interpolated values and compute their standard deviation, but if this pattern is unknown, it can be easily estimated with a simple method. Since there are only two different configurations, the standard deviation from the diagonal and ant-diagonal elements is computed. The set with lower standard deviation is considered as I. For more advanced techniques for CFA pattern detection see [28,29]. We continue with the construction of the required function *f*, which should ideally assign a value of 1 for elements in I and 0 otherwise. The result of applying this function should result in a checkerboard pattern as seen in Figure 4, where 0 would correspond to black squares while 1 corresponds to white ones.

It is assumed that both sets A and I behave as normal random variables with mean 0 and standard deviations $\sigma_{A},\sigma_{I}$ respectively, then having a density defined by (10).

(10) $$\phi_{0,\sigma }\left(x\right)=\frac{1}{\sigma \sqrt{2\pi }}e^{-\frac{x^{2}}{2\sigma^{2}}}.$$

The Gauss Error Function defined in (11) and the Complementary Error Function, given by erfc(x)=1−erf(x), will be useful in the definition of the required function.

(11) $$\mathrm{erf}\left(x\right)=\frac{2}{\sqrt{\pi }}\int_{0}^{x}e^{-t^{2}}dt.$$

The Complementary Error Function is related to the normal distribution function with zero mean and deviation σ, denoted $\Phi_{0,\sigma }$, by (12)

(12) $$\Phi_{0,\sigma }\left(x\right)=\frac{1}{2}\mathrm{erfc}\left(\frac{-x}{\sigma \sqrt{2}}\right).$$

From this relationship, and observing that erfc acquires the value 1 when x=0, and decreases rapidly as *x* grows, we decide that this function is well suited to perform the classification, since, by hypothesis, the errors follow a normal distribution and those elements in I that are estimated with greater precision will get values very close to 1. Finally, the probability function is defined with (13).

(13) $$f\left(x\right)=\mathrm{erfc}\left(\frac{\left|x\right|}{\sigma_{I}\sqrt{2}}\right).$$

**Figure 5:**
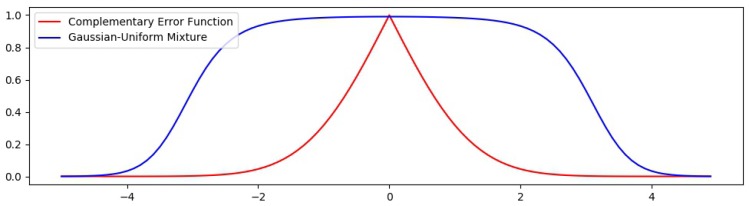
Comparison of the Complementary Error Function and the Gaussian-Uniform Mixture densities with σ=1.

Figure 5 show a comparison between the probability function used in the EM-step, which is also used to provide the probability map used in [16] and the function defined here from the erfc. It is evident that the function *f* discriminates pixels that do not belong to I more precisely, since the curve decays faster as error grows. Experimental evidence supporting the improvement of the classification with this function will be provided in Section 5.

When applying *f* to the error matrix, it is expected that the obtained matrix resembles the 0–1 pattern seen in Figure 4. It will be seen in experimental results discussed in Section 5 that in fact this is the case provided that the hypothesis about residuals distribution is true.

For the final step, the highest frequency coefficient of the DCT coefficients is established as a similarity measure between the ideal 0–1 pattern and the pattern extracted from the CFA artifacts.

### 4.2. Discrete Cosine Transform

The DCT is widely used in image processing and is a very popular tool used for image compression [30]. There are several definitions for the DCT, depending on the way in which a finite sample of a signal extends to create a periodic signal in the domain of the natural numbers [31]. For the proposed method, we have decided to use the first variation of the DCT, denoted DCT-I, since most of the coefficients are zero. Let *X* be a vector of size *B*. The one-dimensional DCT-I transform is given by (14).

(14) $$y_{u}=x_{0}+{\left(-1\right)}^{u}x_{B-1}+2\sum_{i=1}^{B-2}x_{i}\cos\left(\frac{\pi ui}{B-1}\right).$$

The bidimensional DCT-I can be easily computed by applying (14) on all rows of the matrix, and subsequently on the columns of the data matrix.

To show how useful is DCT-I for our purpose, it is applied on a matrix $X=(x_{ij})$ of size B×B, with *B* even, defined by

(15) $$x_{ij}=\left\{\begin{array}{cc} 1 & \mathrm{if}\;i+j\;\mathrm{is}\;\mathrm{odd}\\ 0 & \mathrm{otherwise} \end{array}\right.$$

The *i*-th row of *X* for *i* odd is always a vector of the form $X_{i}=\left(1,0,\cdots ,1,0\right)$. The result of applying the DCT-I on $X_{i}$ is the vector $Y_{i}=\left(y_{0},\ldots ,y_{B-1}\right)$ defined by

(16) $$y_{v}=\left\{\begin{array}{cc} B-1 & \mathrm{if}\;v=0,\;B-1\\ 0 & \mathrm{otherwise} \end{array}\right.$$

On the other hand, the *i*-th row of *X* for *i* even is the vector $X_{i}=\left(0,1,\ldots ,0,1\right)$. In this case, the result of DCT-I is

(17) $$y_{v}=\left\{\begin{array}{cc} B-1 & \mathrm{if}\;v=0\\ -(B-1) & \mathrm{if}\;v=B-1\\ 0 & \mathrm{otherwise} \end{array}\right.$$

To continue with the computation for the 2D DCT-I, the obtained vectors $Y_{i}$ are considered as rows, defining the matrix

(18) $$Y^{\prime }=\left(\begin{array}{cccc} B-1 & 0 & \ldots & B-1\\ B-1 & 0 & \ldots & -(B-1)\\ \vdots & \vdots & \ddots & \vdots \\ B-1 & 0 & \ldots & -(B-1) \end{array}\right).$$

Finally, (14) is applied on the columns of $Y^{\prime }$, obtaining the 2D DCT-I transform on *X*, given by $Y=(y_{uv})$ where

(19) $$y_{uv}=\left\{\begin{array}{cc} 2{\left(B-1\right)}^{2} & \mathrm{if}(u,v)=(0,0),(B-1,B-1)\\ 0 & \mathrm{otherwise} \end{array}\right.$$

To avoid obtaining values that depend on the size of the selected block, the coefficients $y_{u,v}$ are normalized as shown in (20).

(20) $$y_{u}^{\prime }=\frac{y_{u}}{2(B-1)}.$$

It is worth mentioning that DCT-II is more widely used in practice. Many digital image analysis tools have implemented DCT-II by default. Experimental results show that our detection technique is not affected by using one definition or the other. Thus, for simplicity, we refer to any of these definitions as DCT in what follows.

**Figure 6:**
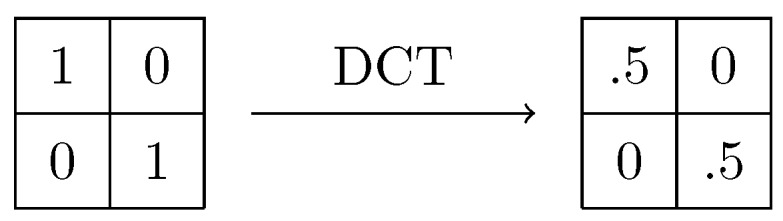
Applying DCT on a 2 × 2 matrix.

In general, by considering small blocks from a big image, the values of the pixels in the block are expected to have a small variation. This means that low frequency coefficients will be higher, which is useful for image compression. A similar approach is required for the ongoing analysis. However, in the proposed method the value of the highest frequency component is considered, since we expect errors to have big differences at adjacent positions. In Figure 6 a block of size 2 × 2 can be observed as a result of applying the the DCT-I on the matrix with values 0 and 1 as defined before.

In a more general fashion, if DCT coefficients, as defined in (20), are computed in a block with alternating values of 0’s and 1’s of size B×B, the values will be 0 except for those at positions (0,0) and (B−1,B−1), whose values will be 0.5 in each case. Although these values are not obtained in practice, relatively high values have been observed in the experiments carried out. As it will be shown in the Section 5, these values will suffice to detect tampering.

### 4.3. Analysis on Red and Blue Bands

**Figure 7:**
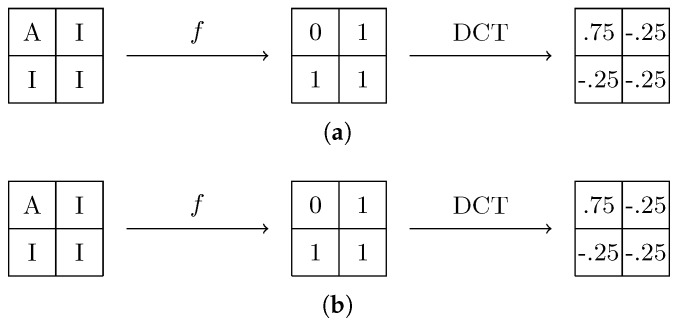
Resulting DCT ocefficients for 2 × 2 blocks of (**a**) red channel and (**b**) blue channel samples.

To provide more accurate results, an analogous analysis can be performed on the red and blue channels. The bidimensional DCT values for a B×B block for the red and blue channel can be seen in (21) and (22) respectively. The 0–1 matrix pattern for a 2 × 2 block is shown in Figure 7a for the red channel and Figure 7b for the blue one.

(21) $$y_{uv}^{\left(r\right)}=\left\{\begin{array}{ccc} 3/4 & \mathrm{if}\;(u,v)= & \left(0,0\right)\\ -1/4 & \mathrm{if}\;(u,v)\in & \{(0,B-1),(B-1,0),\\ & & \left(B-1,B-1)\right\}\\ 0 & \mathrm{otherwise} \end{array}\right.$$

(22) $$y_{uv}^{\left(b\right)}=\left\{\begin{array}{ccc} 3/4 & \mathrm{if}\;(u,v)= & \left(0,0\right)\\ 1/4 & \mathrm{if}\;(u,v)\in & \left\{(0,B-1),(B-1,0)\right\}\\ -1/4 & \mathrm{if}\;(u,v)= & \left(B-1,B-1\right)\\ 0 & \mathrm{otherwise}. \end{array}\right.$$

Analysis of these coefficients can be useful to provide better results in our detection technique. The main problem to be addressed on these channels is that interpolation methods can be way more complex to avoid aliasing and color artifacts, since information other than acquired pixels from the same band is regularly used. To generate a useful probability map better approximation methods must be employed, but estimating using more advanced demosaic algorithm is also helpful, as will be seen in Section 5.

## 5. Experimental Results

To evaluate the proposed method, the analysis has been performed on 165 images gathered from the dataset provided in [6]. The analysis has detected correctly modifications in 158 of them. However, in some cases detection has been partial. Unfortunately, some untampered images have been identified as modified, mainly because some of them show very smooth areas, resulting in very small errors and anomalous values of the DCT coefficient for the highest frequency, which is almost zero as well. As far as we know, this error might affect most of the analysis based on CFA artifacts.

Though four different devices are included within the dataset, only three of them have been considered for the experiments: Nikon D90, Nikon D7000 and Sony A57, containing 55 images per device. The described method has failed in the case of the Canon 60D camera. In this case, the difference between distributions of acquired and interpolated residuals is not observed, and the initial hypothesis is not fulfilled. This may be due to a subsequent smoothing process performed by the device, since specifications of this camera say that this model has an RGGB pattern.

The Python’s libraries OpenCV [32] and SciPy [33] have been used to implement the technique here exposed. As mentioned in Section 4.2 DCT-I has been used for this implementation, which is provided by the SciPy lib. OpenCV is used primarily for image reading and heatmap creation. Histograms have been created using Matplotlib library [34]. The details and main results of the experiments are discussed next.

**Figure 8:**
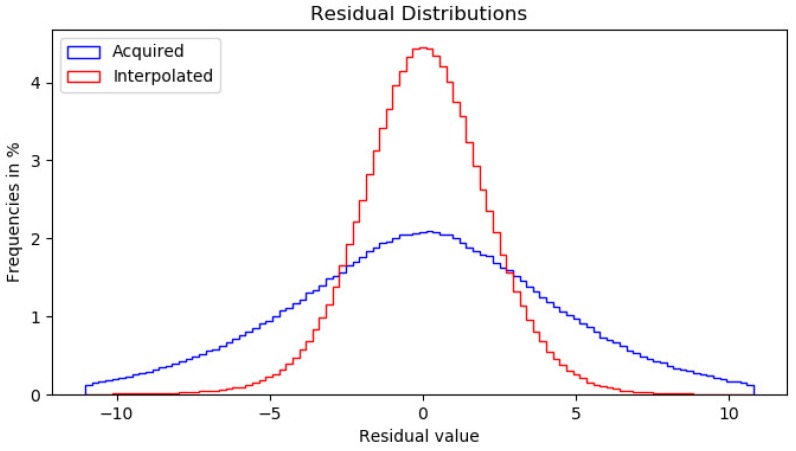
Residual frequencies for interpolated and acquired residuals.

The forensic analysis has been performed as follows. First, for each image the estimation of the interpolation kernel was made using OLS. In this case the estimation is performed with N=2 neighbors, resulting in 12 independent variables. It is verified that residuals from I and A have suitable distributions, as it can be seen in Figure 8. In both cases the distinctive Gaussian bell shape of the normal distribution can be observed. It can be concluded that the image has been interpolated from a Bayer Filter of the form RGGB (or possibly BGGR), so it is possible to continue with the analysis.

**Figure 9:**
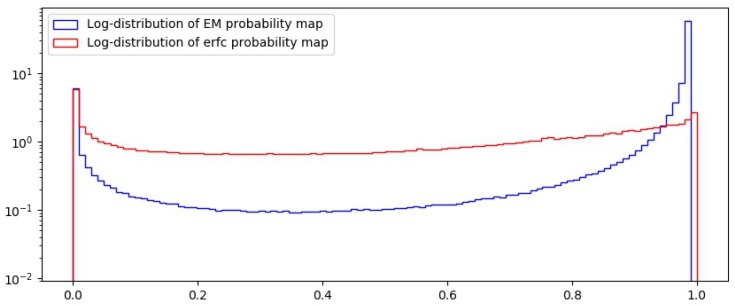
Frequencies for probabilities of interpolated and acquired pixels.

Subsequently, the probability map is calculated by applying the Complementary Error Function. The amount of elements with probability values closer to 1 in the interpolated elements is far greater than those in the acquired set. This will become evident once the DCT is applied on blocks of the image. We have selected B∈{2,4,8,16} to evaluate how well our method performs in finer and broader blocks, allowing us to detect small modifications. To provide a practical argument of the performance of the probability map here described, we show in Figure 9 a comparative of the frequency distribution in logarithmic scale, resulting from the application of the probability functions defined by (1) and (13). The former is used in the EM algorithm proposed in [16] and its distribution shown in blue. The later, shown in red, is the map proposed in the present work. We appreciate that erfc is useful to better classify acquired from interpolated pixels, since roughly half of them are give a value less than 0.5. This does not happen with the mixture of probabilities approach, that accumulates more pixels in the interpolated set by assigning a probability of more than 0.5 to a higher percentage.

**Figure 10:**
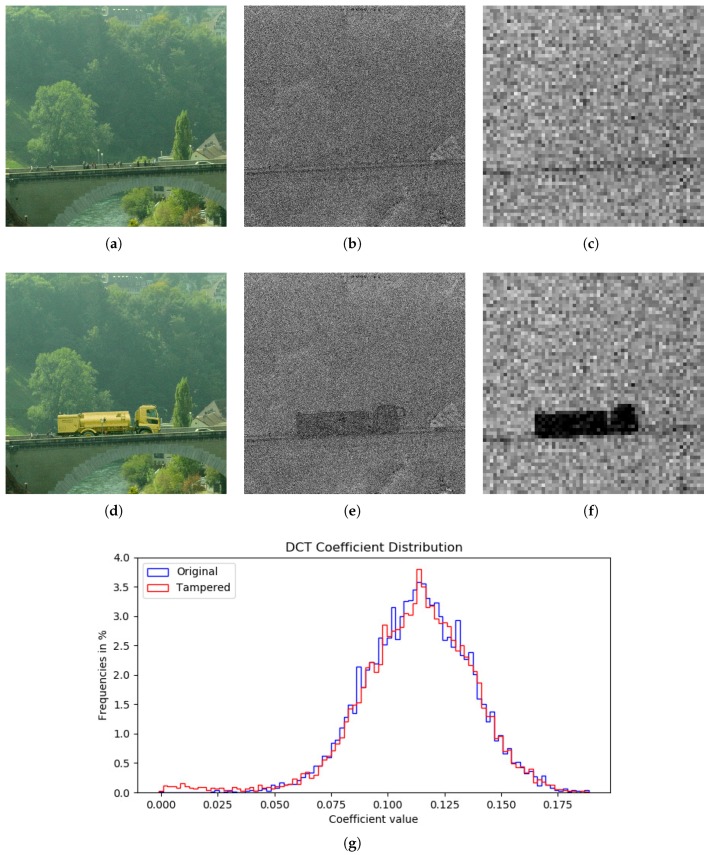
Probability maps and heatmaps in black and white of (**a**) original image vs. (**d**) tampered image. Subimages at (**b**,**e**) correspond to their probability map and (**c**,**f**) show a heatmap for the result of applying DCT on blocks of size 16. Histogram in (**g**) shows the distribution of DCT coefficients for original and tampered images. A broader left tail can be seen for the tampered one, corresponding to low values and exhibiting a possible modification.

Results of the analysis made to one of the tampered images from the dataset and the data obtained on each step of the process can be observed in Figure 10. Subimage Figure 10b corresponds to a picture taken with the Nikon D7000 camera and has been modified by adding new elements in it.

The probability map computed on the estimation is shown in Figure 10c,d. A darker area indicating the tampered zone becomes apparent, though it is weakly observed. Finally a more defined black rectangle becomes visible in the heatmap for DCT coefficients in Figure 10f, showing the outcome of applying DCT on blocks of size B=16.

Bad estimations on edges or areas with texture may cause problems. In Figure 10, dark regions can be visible near the zone of the bridge of the untampered image (Figure 10b,c) because of a bad estimation for the edge. This causes a change in the expected configuration for errors of acquired and interpolated pixels, modifying the corresponding values of the probability map and consequently the DCT coefficients, which are lower. This is not a problem in most of the cases, since tampered areas are commonly bigger and thus more visible after being processed.

For a better appreciation of the outputs of the detection technique, images having a size of 1920×1080 have been cropped to a size of 1080×1080. However the analysis has been performed on the totality of the pictures. Also, the range of heatmaps for DCT coefficients has been adjusted according to their distribution, setting the minimum (black) to the 5th percentile and the maximum (white) to the 95th percentile. With this, the tampered regions are easily perceived.

In Figure 10g, the histogram of the values obtained when applying DCT shows a small concentration of values around 0 in the tampered image. This does not happen in the original image.

**Figure 11:**
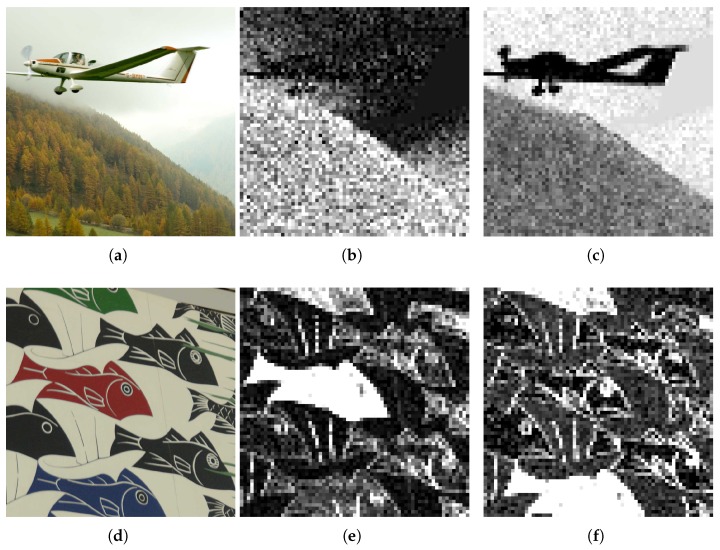
Alternative image estimations. Image (**a**) is tampered by adding a new element. DCT coefficients are not useful to discover the tampered region correctly when estimating every pixel (**b**). However, if acquired pixels remain unchanged, the tampered area is perfectly visible (**c**). Image (**d**) shows a colorization tampering. Detection is achieved by estimating with constant-hue demosaicing and applying the test on the red and blue channels. Results are shown in (**e**,**f**) respectively. Brighter zones show modified objects. This modification is not disclosed with green channel analysis.

In addition to the process here explained, a different approach has been tested for the estimation process. Since it is assumed previous knowledge of the Bayer Filter configuration, the estimation is computed by leaving the acquired pixels unchanged. The kernel approximation process is the same as proposed before, but it is not applied it on the acquired data. This has been useful to highlight tampered areas surrounded by smooth areas, as shown in Figure 11.

Finally, another approach has been taken to detect color manipulations. Instead of an OLS estimation for the interpolation kernel of red and blue channels, we directly perform a demosaicing method on the Bayer Filter. For the study here presented, we have chosen to estimate the image by using the edge-oriented constant-hue interpolation, and results have proved useful. Figure 11 shows that this approach has detected successfully the change in color in the red, green and blue fishes. This is not shown with the green channel analysis.

### 5.1. Detected Manipulations

Some of the modifications that have been adequately detected with our technique are listed below.

**Figure 12:**
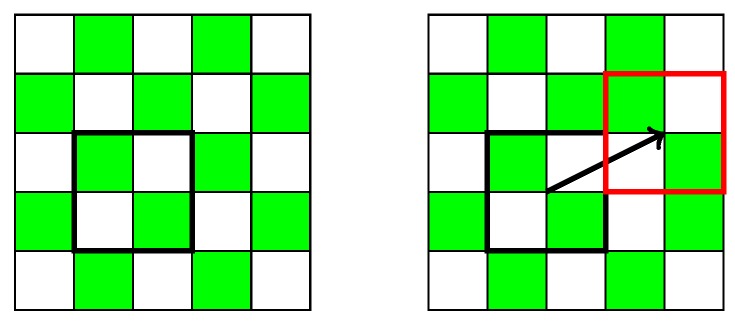
Tamper of green channel pattern by a copy-move modification. The area inside the black square is copied and moved, breaking the CFA pattern within the red square.

- Copy-move. If the green channel of the image is altered in a similar way to what is shown in Figure 12, then the DCT coefficients will exhibit the change in the pattern. The coefficient for the highest frequency will become negative in the affected zone, resulting in an accumulation of values below zero. However, if the green pattern is not broken, the manipulation will not be exposed.
- Blurring filters. It has been possible to locate the use of blur filters, such as the Gaussian and Median filters among others. In the smoothed section the high frequencies are high-passed, and the difference between the value of the deviations $\sigma_{I}$ and $\sigma_{A}$ disappears, which makes the modification visible after the analysis.
- Insertion from external sources. Detection occurs mainly for two reasons. As explained in the copy-move detection, the characteristic grid of the green color can be lost when inserting an image from an external source. However, a modification can also be detected by adding elements obtained by a different interpolation. In this case the distribution of residuals will be different in the analyzed area, unless the new object comes from the same camera model.
- Geometrical transformations. The effect of resampling under geometric transformations, like resizing and rotation, introduces new correlations. In the case of copy-move forgeries involving geometrical transformations, the area will exhibit a different pattern. If the transformation is carried out in the totality of the image, the CFA artifacts will be lost, but using only 1 coefficient is not enough to detect the specific transformation applied.
- Color change. We have tested the analysis using the red and blue channels on the same set of images. In general, examination for green channel tampering has outperformed the other two, but we have seen that color change manipulation might be detected only by red or blue channel analysis as seen in Figure 11.

### 5.2. Results of Dataset Analysis

Even though our algorithm has been successful on most of the cases, we have observed some drawbacks including both, false positives and false negatives. By adding elements in the image either from external sources or from the image itself, the grid of the green channel is not always altered. If the source also comes from the same device, it may not generate any inconsistency in the distribution of the residuals. We have also noted that areas containing extreme color registers create very smooth areas, which can be detected as a modification carried out by applying a smoothing filter. A more complete detection process should use some other tools that address specific tampering methods, as mentioned in Section 2. It would be preferable to choose tools that do not depend on the same assumptions here required, which involves detection of CFA pattern inconsistencies, since probability of failure will be high. Also detection of smooth areas that in many cases cause false positives should be addressed carefully, since smoothing image filters can be erroneously discarded. We have not dealt with this problem yet precisely because of this reason.

We present a summary of the results of the analysis conducted on the dataset aforementioned in Table 1. In most of the cases it is evident that tamperings have been carried out by performing a simple copy move or an image splicing, this is, by adding an image from an external source. However, no additional information has been found other than camera models and the software used to modify the images (GIMP and Affinity Photo). Thus, a third row has been added when the tampering method cannot be decided precisely. A total of 165 pictures have been analyzed and in 24 of them no evidence of tampering could be found properly, achieving an accuracy of 86% for the detection of a modification, although only partial in some cases. In many images, it has been observed that more than one tampering method has been applied, thus the total count of detected tamperings is 152 (total or partial), but the amount images detected as tampered is 141. For copy-move, we get 53 out of 65 detections for an accuracy of 82%. It was observed that a copy move followed by a rotation, reflection or resizing is easily detected, since the pattern is broken with this transformations, though few of these were found. A total of 70 Images contained splicing tamperings, and we were able to detect 68 of them for an accuracy of 86%. Finally, unknown modifications comprised 31 of the samples, of which 9 of them could not be detected, thus achieving and accuracy of 76%. Identifications providing at least 70% of the tampered area are shown on the left section of the “Identified” column. On the right, thewe show the total count for smaller detections. A big amount of false positives is shown in every case, which are mostly because of bright or dark sections in the pictures. Most of these problems are easily detected from the image, but are difficult to avoid programmatically.

**Figure 13:**
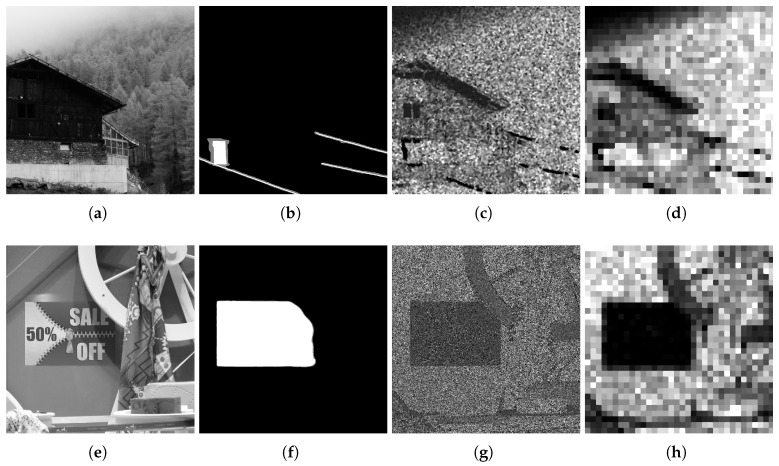
Misbehaviour of detection depending on the block size. The first column shows the analyzed images. In the second column, images (**b**,**f**) highlight the modifications in white. Images (**c**,**g**) represent the result of the analysis on the images with a block of size 2 × 2. Finally, images (**d**,**h**) exhibit the performance on blocks of size 32 × 32.

Evidence shows that the algorithm behaves better when effected on blocks of size 16×16, since modified areas are highlighted more consistently. However, if modifications are of smaller size. A small block could outperform the bigger ones. We picture an example of this case in Figure 13c, where a thin area is modified. In this case, for B=2 the analysis exhibits the affected area, but for B=32 some black areas, arising from smooth sections of the image, can be confused as modified while the affected area is not shown properly. On the other side, Figure 13g,h show how a big tampered area is better detected with B=32.

Table 2 shows how the choice of *B* can affect the performance of the test, which mostly depends on the size of the alteration.

## 6. Conclusions and Future Work

A technique developed to analyze images obtained from high-end digital cameras has been evaluated. The experiments were performed using 165 images of a public dataset to test the reliability of the proposed technique. We opted for this option, since the production of ad hoc images using basic interpolation methods, such as bilinear and bicubic, among others, results are very accurate, but do not reflect the usefulness of the proposed technique in real life cases. In the results of the experiments 86% accuracy was obtained in the detection of manipulated images where CFA artifacts were found, being a very satisfactory result.

A great advantage compared to other methods briefly discussed in Section 2 lies in the fact that it detects a wide range of modifications, as it has been argued in Section 5. Additionally, in comparison with related techniques that take advantage of the CFA artifacts exposed by interpolation, the method here detailed is simple can be considered as a quick and lightweight alternative, thus it can be considered as the first resource for image forensic analysis, before performing more computational complex methods and specialized techniques.

We have seen that better interpolation methods can provide more accurate results, specially in the case of blue and red color channels. This can be caused because many demosaicing algorithms are constructed to prevent image artifacts related to edges. Then, a picture with few edges and big smooth areas, as it is commonly the case, would use methods that behave similarly to most known demosaicing techniques.

Future research will consider different CFA configurations and color spaces other than RGB. Also automatic recognition of tampering is desirable to provide a more powerful tool for forensic analysis, considering the shape and density of the detected area, as well as the inconsistencies in the DCT values as shown in histograms of Figure 10.

## Figures and Tables

**Table 1:** Detection per tampering method and source.

	Tampering Method	Identified		Not Identified	False Positives
		≥70%	<70%		
Nikon D7000	Copy-move	16	4	2	11
	Splicing	25	2	5	
	Others	8	5	0	
Nikon D90	Copy-move	8	13	4	13
	Splicing	19	4	2	
	Others	4	4	0	
Sony A57	Copy-move	9	3	6	14
	Splicing	13	5	5	
	Others	10	0	0	
	Total	112	40	24	38

**Table 2:** Detected images per block size.

Block Size	2×2	4×4	8×8	16×16	32×32
Detections (out of 165)	126	132	138	139	138
